# The Science and Politics of Colorectal Cancer Screening

**DOI:** 10.1371/journal.pmed.0030036

**Published:** 2006-01-03

**Authors:** Geir Hoff, Michael Bretthauer

## Abstract

Hoff and Bretthauer discuss the scientific and political controversies surrounding the introduction of a national screening programme for colorectal cancer.

Politicians in developed countries have secured a certain standard of health care for the sick as well as vaccination programmes to reduce the risk of illness. For some of these countries, cancer screening is next on the agenda, with breast and cervical cancer screening programmes at the top of the list. When it comes to colorectal cancer (CRC), randomised trials of screening with the faecal occult blood test (FOBT) have shown that such screening reduces CRC incidence [1] and mortality [1–3]. Indirect evidence suggests that endoscopic screening (flexible sigmoidoscopy and colonoscopy) may be far more effective, but results from large-scale randomised trials are still awaited [4–7].

In the United States, CRC screening, using any of these screening modalities, has been recommended for many years [8–10]. The European Union now encourages its member states to initiate CRC screening, provided that it is done in an organised fashion and linked to quality assurance programmes [11]. Although the EU guidelines point out that only FOBT screening has been proven in randomised trials to have an effect on CRC, some member states (Poland, Germany, and Italy) have gone straight for “gold standard” colonoscopy screening without evidence from randomised trials [12,13].

The ideal screening method has not yet been found, and the future may see more use of promising screening modalities such as stool-based DNA tests [14] and virtual colonoscopy [15]. At present, however, the fact that these newer methods are still at an early stage of development may only serve as an excuse for politicians to “wait and see”, and thus further postpone a decision on CRC screening. In this essay, we discuss the scientific and political controversies surrounding the introduction of a national CRC screening programme. We argue that it is in the best interest of governments to fund well-conducted randomised trials that can guide their decision making.

## Public Awareness of CRC

Enthusiastic health professionals often find politicians and health authorities too slow to respond to the considerable evidence in favour of CRC screening [13], and they may take on a political role themselves in promoting their good cause. In addition, a number of celebrities—including Ronald Reagan, Katie Couric (an American television personality), and Lynn Faulds Wood (a British television presenter)—have helped to raise public awareness of CRC ([16]; http://www.bowelcancer.tv/cgi-bin/page.pl). His Holiness, the late Pope Paul John II, promoted CRC screening by being the first patron of the International Digestive Cancer Alliance, with strong support from United States Senator Hillary Clinton [17].

But while there is growing public awareness of CRC, there has been disturbingly little public discussion about the scientific pros and cons of CRC screening compared with what some countries have seen for breast cancer screening. One of the main reasons for nonparticipation in CRC screening has been that people are not convinced that they will gain anything by participating [18]. Similarly, the failure of some physicians to recommend screening may reflect a general need for more convincing evidence and a convincing presentation of such evidence. Women being offered mammographic screening for breast cancer have expressed a particular need for information about logistics as well as about false positive and negative tests, and they want to play an active part in decision making on screening [19]. This need for balanced information on screening applies to other types of cancer screening and should be the “standard of care” for future cancer screening awareness campaigns.

By failing to recommend screening, physicians representing the medical profession may appear divided in their view to screen or not to screen, and undecided on which method to use, although the health problem of CRC is recognised by all. In many countries, CRC is one of the two most frequently encountered cancers for men and women collectively. Five-year survival is only about 50%, increasing to 80% if diagnosed at an early, asymptomatic stage [20]. Thus, early diagnosis and treatment is the obvious key to improved cure and survival.

Some enthusiasts initially recommending national screening programmes with continuous quality assurance now accept opportunistic screening (i.e., screening done outside of a national screening programme), since “public health policy has not yet included population CRC screening” [13]. On the other hand, many doctors feel that there are still important questions to be answered before implementing national screening programmes. Besides, some are concerned that not enough is being done for patients who need basic health care rather than preventive measures.

## How Good Is the Evidence for Screening?

So what kind of evidence can we offer to increase professional enthusiasm for CRC screening? Do we only have the support of celebrities and little else?

FOBT is the only screening method submitted to adequately designed randomised trials, which showed 15%–33% mortality reduction from CRC after 8–13 years' follow-up [1–3]. The relative mortality reduction was significant in all three trials, proving without doubt that “FOBT screening does work”. But how good is this in absolute, not relative, terms? With 5% lifetime risk of CRC and 50% mortality from the disease, the risk of dying from CRC is 2.5% without FOBT screening. With biennial FOBT screening, this may be reduced by 23% for those attending, i.e., reduced by 0.6% to 1.9% if attendance is 100%. About 98% of us will die from something else. How do you sell such figures both to doctors and to the public?[Fig pmed-0030036-g001]

**Figure pmed-0030036-g001:**
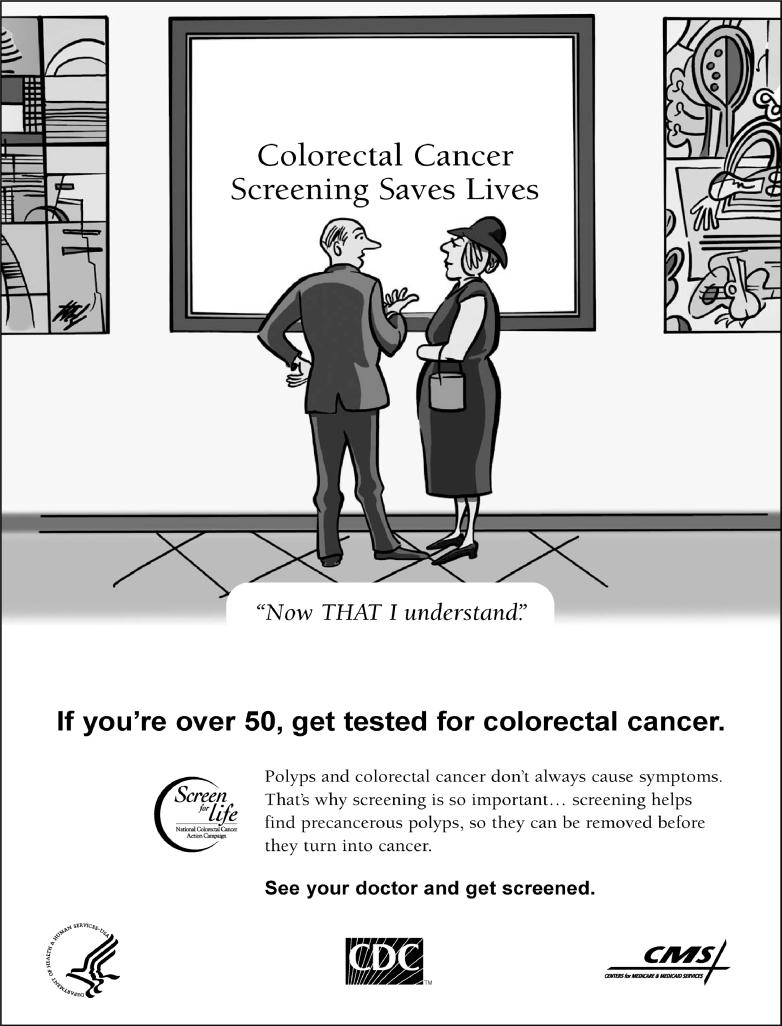
In the US, the CDC (Centers for Disease Control and Prevention) is promoting CRC screening for people over 50 years old

In contrast to FOBT, we only have case-control studies to support the usefulness of endoscopy screening, and such studies are particularly prone to overestimate the effectiveness of screening tests when compared with randomised trials [21]. The first reports from the four ongoing randomised trials on flexible sigmoidoscopy screening are expected over the course of the next several years [4–7], while similar randomised trials on colonoscopy screening have not, so far, been launched. Mortality reduction by colonoscopy screening is expected, but not proven, to be much higher than the 23% for those attending FOBT screening, in addition to an incidence reduction expected through polypectomies.

There has been some concern about a possible excess number of non-CRC deaths in the screening group in CRC screening trials [22,23]. In addition, a recent 17-year follow-up of the Danish FOBT study showed that the significant reduction in mortality seen after about ten years could no longer be observed, probably due to poorer attendance with an increasing number of screening rounds [24]. Since high attendance rates are crucial for the success of screening with the FOBT, the best choice among the present CRC screening options may differ between countries and cultures, thus indicating a need for research on CRC screening referable to the target population. Furthermore, there is only limited knowledge about what impact an increasing menu of screening services might have on the responsibility people take for their own health. For example, patients may abandon healthy lifestyles (exercising, eating plenty of fruit and vegetables, giving up smoking) if they believe that screening will pick up cancers at an early stage.

## How Far Should We Compromise on Scientific Proof?

The crucial question is the following: how much evidence (and at what level in the well-known hierarchy of evidence [http://www.shef.ac.uk/scharr/ir/units/systrev/hierarchy.htm]) is required before implementing national screening programmes? The World Health Organization guidelines for screening request evidence for mortality and morbidity reduction from high-quality randomised controlled trials before starting any population screening programme [25]. In other fields of medicine, such as pharmacotherapy, randomised trials have been a standard requirement for introducing any new drug on the market.

Many health services have admittedly been introduced based on much weaker evidence than we now have for CRC screening. But historic substandards should be no argument for turning a blind eye to the need for the best-quality evidence or for allowing health policy decisions to be based more on ideology and convictions than science. Also, the concept of pushing a screening service onto presumably healthy people is quite different from establishing a health service that people seek when they feel sick. The level of evidence should therefore be more foolproof for screening services than for other services, and the evidence should be able to withstand debates such as the one surrounding mammography. High-quality evidence on CRC screening would also help to unite the somewhat divergent professional views on this intervention.

## The Finnish Model

We need to recognise that some countries feel that the introduction of national CRC screening programmes is an urgent matter. Ideally, these should be rolled out in a stepwise, randomised fashion, just as Finland has done (see Box 1), allowing evaluation of its FOBT screening programme after five years before deciding what their next step of action should be [26]. The people behind the Finnish strategy deserve credit for persuading their politicians to choose this cautious, stepwise model, and the politicians and health authorities deserve credit for listening.

**Box 1. Introducing a CRC Screening Programme: The Finnish Approach**

In 2004, Finland launched a randomised, stepwise introduction of CRC screening using FOBT (biennial, unrehydrated Hemoccult-II). For the first six years of introduction, each age cohort is randomised to screening or “care as usual” (no screening) at age 60–64 years. In six years, 50% of the entire target population will be covered. This provides an opportunity to evaluate the programme after five years and to further adjust the screening strategy or to implement FOBT screening to all Finnish citizens aged 60–69 within the ensuing five years. Presently, representatives from the five Nordic countries are collaborating to develop the Nordic Initiative on Colorectal Cancer, an investigator-initiated protocol with an aim to further the scientific knowledge of CRC screening.

In the Finnish model, half of each age cohort is randomised to screening or no screening. The Finnish model must have required a lot of explanation to authorities that this approach was clearly the best way to proceed. It was, of course, risky for politicians to voluntarily throw away half (or more) of their target candidate supporters by declaring, in essence, “We believe in CRC screening, but aren't sure about it, and half of you will be offered screening while the other half will not”.

In contrast to the Finnish approach, there have been examples of political decisions to simply screen everyone or none at all, i.e., to reject the idea of large-scale randomised trials. This rejection may leave health-care providers and doctors with a very difficult choice: either to go straight for national screening or to reject the political offer and wait for a new set of politicians who may understand the need for further randomised trials.

## Short-Term Gain, Long-Term Pain?

For those who have experienced CRC or have been close to someone suffering from it (a considerable number in Westernised societies), any proven benefit from screening will do. Furthermore, CRC screening may appear politically attractive and tempting to politicians when campaigning for a general election. And cost–benefit estimates have even suggested that such screening may be economically sound and at least as cost-effective as established national screening programmes (for breast and cervical cancer) [27].

But politicians should be concerned about whether CRC screening is the right way to spend available resources and taxpayers' money for the benefit of individuals, families, employers, and the community. There are still few data to guide decisions about CRC screening. It is hard to understand why politicians are reluctant to invest in randomised clinical trials, since the results of these will equip them to make better political decisions in the end.

The alternative to taking a firm grip on development toward national programmes through the funding of large, well-designed randomised controlled trials seems to be uncontrolled out-of-pocket expenditure on sporadic screening, which cannot generate any real evidence about the effectiveness of screening. Any country considering national CRC screening can surely afford to do its own randomised trials, but time is running out. Could it be that none of the providers really want to know, but prefer to make decisions by convictions and “gut feelings” for short-term gain? For a screening programme to survive over time, it has to deliver according to expectations. If expectations are not based on trials referable to the target population, any programme will be vulnerable to devastating debates, such as those surrounding mammographic screening seen in some countries.

Health policymakers must also remain sceptical of the role of celebrity endorsements. Communication on complex decisions such as cancer screening, with an aim to inform rather than persuade, is not an obvious task for celebrities [28]. Likewise, it is not an obvious task for doctors to take on the role of politicians. Instead, politicians must accept the need for more science in their decision making. We need more objective facts, relevant to the target population, to be communicated—and not personal convictions that doctors, doctor-politicians, and politicians only present as “facts”. To paraphrase former US President Franklin Delano Roosevelt: “Look to Finland”.

## Abbreviations

CRC: colorectal cancer
FOBT: faecal occult blood test
